# Induction of T-cell mitogenic unresponsiveness by recombinant human granulocyte colony-stimulating factor (rHuG-CSF)

**DOI:** 10.1054/bjoc.1999.1184

**Published:** 2000-04-03

**Authors:** S Rutella, C Rumi, S Sica, G Leone

**Affiliations:** Department of Haematology, Catholic University Medical School, Largo A Gemelli 8, Rome, 00168, Italy


*British Journal of Cancer* (2000) **82**(9), 1610–1614
© 2000 Cancer Research Campaign
DOI: 10.1054/ bjoc.1999.1184, available online at http://www.idealibrary.com on IDEAL®

Letters to the Editor

### Induction of T-cell mitogenic unresponsiveness by recombinant human granulocyte colony-stimulating factor (rHuG-CSF)

**Sir**,

We read with interest the article by Reyes et al (1999) describing a transient suppression of lymphocyte proliferative response to polyclonal mitogens in patients affected by breast cancer and receiving rHuG-CSF to mobilize peripheral blood progenitor cells (PBPC); these variations occurred irrespective of changes in both T-cell subset distribution and lymphocyte ex vivo 'activation status', as measured by expression of HLA-DR and CD25 activation-related antigens.

Data reported by Reyes et al confirm that G-CSF treatment modulates the proliferative response of peripheral blood mononuclear cells to T-cell mitogens in humans. Investigations performed by our group in healthy subjects treated with rHuG-CSF to mobilize PBPC have already demonstrated a humoral-mediated rHuG-CSF-induced suppression of lymphocyte cycling after mitogenic challenge (Rutella et al, 1997*a*; 1997*b*); interestingly, lymphocyte proliferation inversely correlated with increased serum levels of immunoregulatory cytokines, i.e. lactoferrin and interleukin-1 receptor antagonist (IL-1ra), strongly suggesting indirect actions mediated by soluble factors; this hypothesis is further supported by the observation that lymphocytes apparently lack G-CSF receptors (Mellstedt et al, 1999; Hartung, 1999).

We next evaluated the effects of serum collected after rHuG-CSF administration (G-serum) on the proliferation of normal allogeneic lymphocytes (Rutella et al, 1998); when PHA-challenge was performed in the presence of G-serum, lymphocyte cycling was strongly inhibited in a concentration-dependent manner and lymphocytes were arrested in a G0-like phase of the cell cycle, although they up-regulated the expression of early (CD69) and late activation related antigens (CD25, CD71 and HLA-DR), enlarged into blasts and secreted normal amounts of IL-2. Taken together, these features recapitulate the phenotype known as lymphocyte partial activation, which consists of: (1) preserved blast transformation, (2) preserved up-regulation of activation-related antigens, i.e. CD69, CD25, CD71 and HLA-DR, (3) preserved secretion of IL-2 and (4) inability to progress through the cell cycle upon mitogenic stimulation. Lymphocyte partial activation has been regarded as a fundamental mechanism for tolerance induction in T-cell clones and might control the amplification of the immune response (Sloan-Lancaster et al, 1994).

From our data (Rutella et al, 1999) and those by other groups (Hartung, 1999), a new intriguing role of rHuG-CSF as an immune suppressive agent has emerged. The modulation of immune responses might be beneficial after the infusion of rHuG-CSF-mobilized allogeneic PBPC; conceivably, similar incidence and severity of acute graft versus host disease (GVHD), which has been recently reported after allogeneic PBPC compared to bone marrow transplantation, might be explained by a functional alteration of lymphocytes exposed in vivo to rHuG-CSF.

Interestingly, recent studies in a murine allogeneic transplant model demonstrated a G-CSF-induced T-cell polarization toward a type-2 phenotype (Pan et al, 1999), leading to attenuated secretion of inflammatory cytokines from Th2 $CD4^+$ T-cells and to enhanced graft-versus-leukaemia (GVL) activity from Tc2 $CD8^+$ cytotoxic T-lymphocytes (CTL); thus, G-CSF-primed T-cells might: (1) possess a diminished capacity to induce severe acute GVHD and (2) maintain GVL function and efficiently induce apoptosis of leukemic targets through a perforin-dependent pathway.

Investigations are currently ongoing to analyse the regulation of lymphocyte cell cycle machinery after exposure to rHuG-CSF (manuscript in preparation). Whether immune dysfunction will favourably impact on incidence of acute GVHD after allogeneic PBPC transplantation remains to be demonstrated in large series of patients.

*S Rutella, C Rumi, S Sica and G Leone*
*Department of Haematology, Catholic University Medical School, Largo A Gemelli 8, 00168 Rome, Italy*

### REFERENCES

Hartung T (1999) Immunomodulation by colony-stimulating factors. *Rev Physiol Biochem Pharmacol* **136**: 1–164

Mellstedt H, Fagerberg J, Frodin JE, Henriksson L, Hjelm-Skoog AL, Liljefors M, Ragnhammar P, Shetye J and Ostenborg A (1999) Augmentation of the immune response with granulocyte-macrophage colony-stimulating factor and other hematopoietic growth factors. *Curr Opin Hematol* **6**: 169–175

Pan L, Teshima T, Hill GR, Bungard D, Brinson YS, Reddy VS, Cooke KR and Ferrara JLM (1999) Granulocyte colony-stimulating factor-mobilized allogeneic stem cell transplantation maintains graft-versus-leukemia effects through a perforin-dependent pathway while preventing graft-versus-host disease. *Blood* **93**: 4071–4078

Reyes E, Garcia-Castro I, Esquivel F, Hornedo J, Cortes-Funes H, Solovera J and AlvarezMon M (1999) Granulocyte colony-stimulating factor (G-CSF) transiently suppresses mitogen-stimulated T-cell proliferative response. *Br J Cancer* **80**: 229–235

Rutella S, Rumi C, Testa U, Sica S, Teofili L, Martucci R, Peschle C and Leone G (1997*a*) Inhibition of lymphocyte blastogenic response in healthy donors treated with recombinant human granulocyte colony-stimulating factor (rhG-CSF): possible role of lactoferrin and interleukin-1 receptor antagonist. *Bone Marrow Transplant* **20**: 355–364

Rutella S, Rumi C, Lucia MB, Sica S, Testa U and Leone G (1997*b*) Humoral mediated suppression of lymphocyte blastogenesis in healthy donors receiving rhG-CSF. *Eur J Histochem* **41**: 51–52

Rutella S, Rumi C, Lucia MB, Sica S, Cauda R and Leone G (1998) Serum of healthy donors receiving granulocyte colony-stimulating factor induces T-cell unresponsiveness. *Exp Hematol* **26**: 1024–1033

Rutella S, Rumi C, Sica S and Leone G (1999) Recombinant human granulocyte colony-stimulating factor (rhG-CSF): effects on lymphocyte phenotype and function. *J Interferon Cytokine Res* **19**: 989–994

Sloan-Lancaster J, Evavold BD and Allen PM (1994) Th2 cell clonal anergy as a consequence of partial activation. *J Exp Med* **190**: 1195–1205

